# Supplementary material for: Reaction-diffusion in a growing 3D domain of skin scales generates a discrete cellular automaton
Source: Nat Commun. 2021 Apr 23;12:2433. doi: 10.1038/s41467-021-22525-1 (PMC8065134; doi:10.1038/s41467-021-22525-1)
Supplement: Supplementary file 1 — Supplementary Information [file 41467_2021_22525_MOESM1_ESM.pdf]

Nature Communications 2021

Reaction-diffusion in a Growing 3D Domain of Skin Scales Generates a Discrete Cellular Automaton

SUPPLEMENTARY INFORMATION

Anamarija Fofonjka[1,2] & Michel C. Milinkovitch[1,2,*]

1. Laboratory of Artificial & Natural Evolution (LANE), Dept. of Genetics & Evolution, University of Geneva, 1211 Geneva, Switzerland.
2. SIB Swiss Institute of Bioinformatics, Geneva, Switzerland.

*To whom correspondence should be addressed:
Michel C. Milinkovitch,
Laboratory of Artificial & Natural Evolution (LANE),
Dept. of Genetics & Evolution, University of Geneva,
Sciences III, 30, Quai Ernest-Ansermet, 1211 Geneva 4, Switzerland.
Tel: +41(0)22 379 67 85; e-mail: Michel.Milinkovitch@unige.ch
ORCID ID: 0000-0002-2553-0724

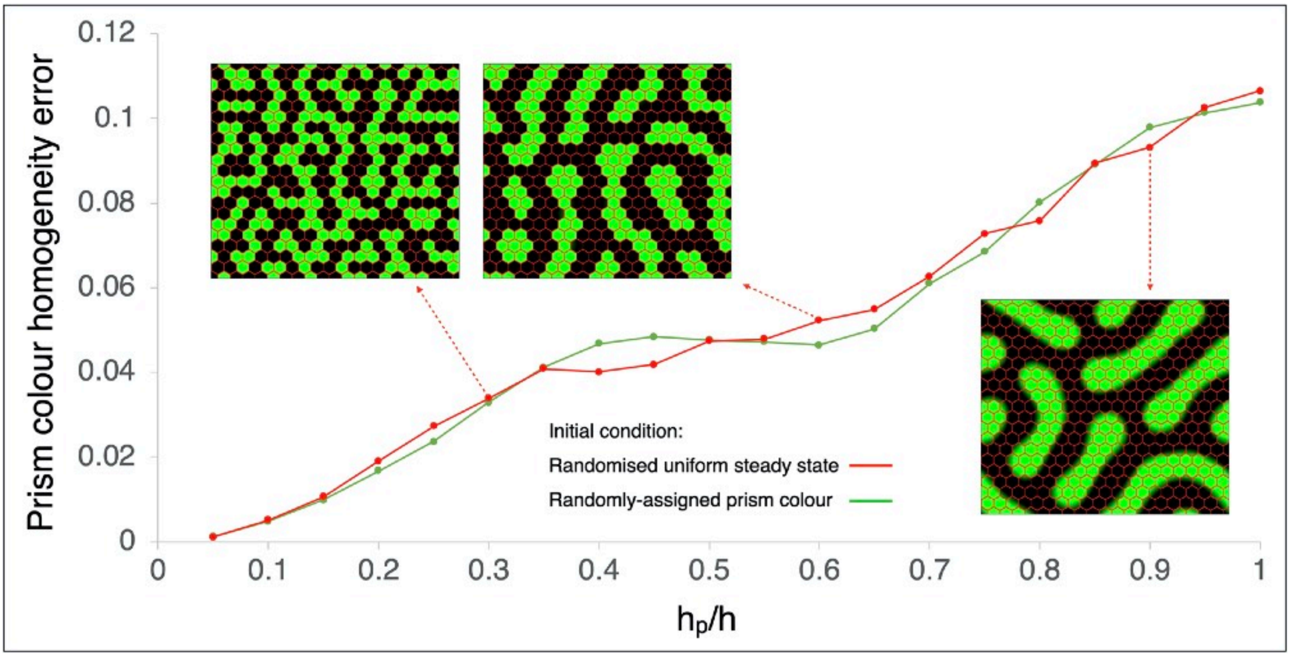

Supplementary Figure 1 | Sensitivity analysis — The prism colour homogeneity error, which measures, at steady state, the deviation from homogeneously-coloured prisms, decreases with decreasing h_p/h ratio. When the inter-prism thickness reaches 30% of the prism thickness ($h_p/h = 0.3$), the chains of black scales tend to exhibit a width of 1 to 2 scales and the prism colour homogeneity error (averaged across all prisms) is below 4%. An error of zero would correspond to each prism having all its simulation grid points of identical colour; see Methods). Green line: simulation starting with random assignment of prism colours: either black (u,v,w)=(1.2, 6.6, 2.3) or green (u,v,w)=(5.3, 0.92, 4)). Red line: simulation starting with all prisms assigned the uniform steady-state colour (u,v,w)=(3.5, 3.0, 3.4) ±1% noise. Inset images show steady-state top views for h_p/h = 0.3, 0.6 and 0.9.

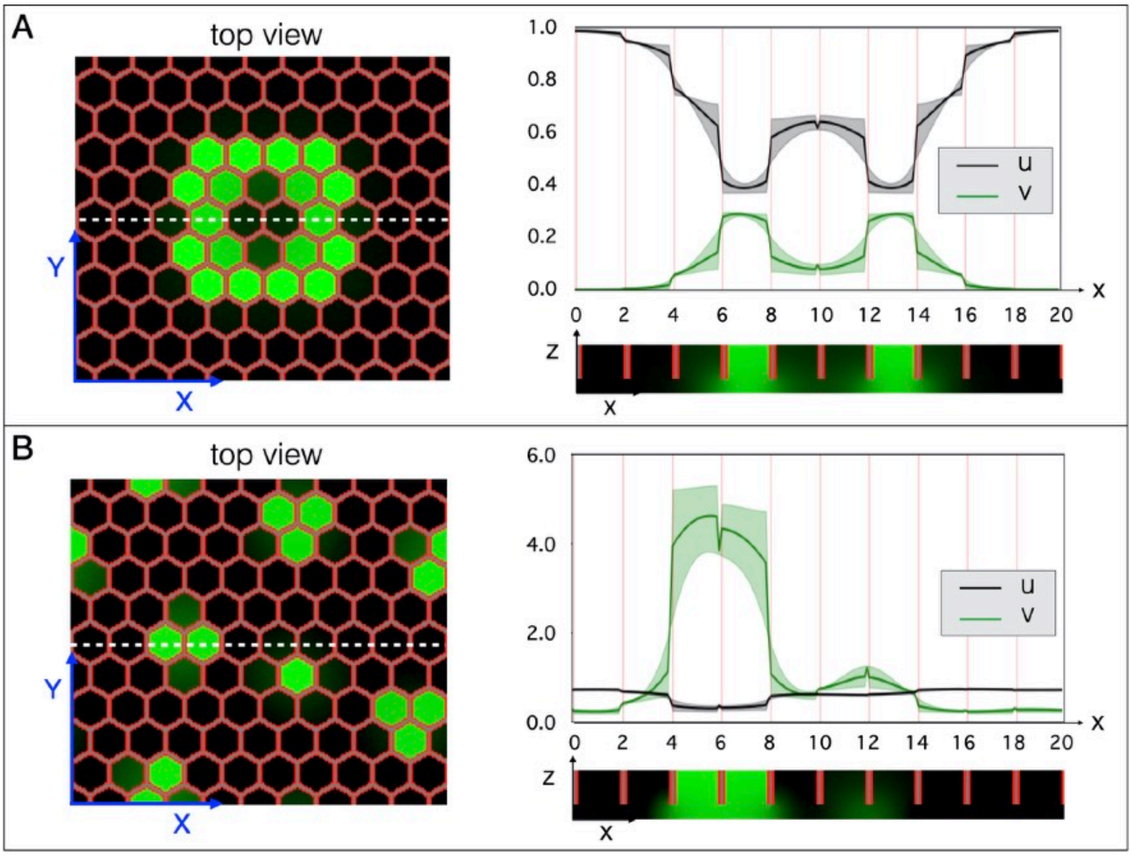

Supplementary Figure 2 I Changing initial conditions in two-component RD models. (**A**) Steady state obtained with the Gray-Scott RD model (ε=0.1, F=0.042, k=0.063, Du = 0.15 and Dv=0.075) with initial condition as in[1]: the entire field is first placed in the trivial state (u,v)=(1,0); second the 100 x 100 mesh point area in the centre of the simulation domain is set to (u,v)=(0.5, 0.25); and third, ±1% random noise is added to break the symmetry of the square. (**B**) Steady state obtained with the Schnakenberg model (a=0.1, b=0.9, γ=1, d=40) with homogeneous initial condition (u,v)=(1,0.9) perturbed with ±1% random noise. In both (A) and (B), the domain size is 200 x 173.

Reference 1: Pearson, J. E. Complex patterns in a simple system. *Science* **261**, 189-192 (1993).

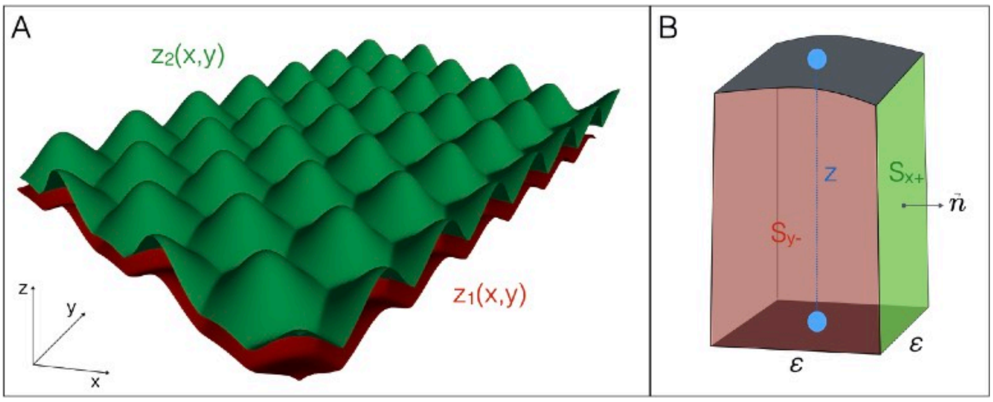

Supplementary Figure 3 | Geometry of large skin patches — (**A**) 3D geometry bounded form below and above by two smooth 2D functions $z_1(x,y)$ and $z_2(x,y)$. (**B**) Elementary volume V centred in (x,y) of width and depth ε, thickness $z = z_2(x,y) - z_1(x,y)$ and bounded by the surfaces S_{x-} and S_{x+} in the x direction and S_{y-} and S_{y+} in the y direction.

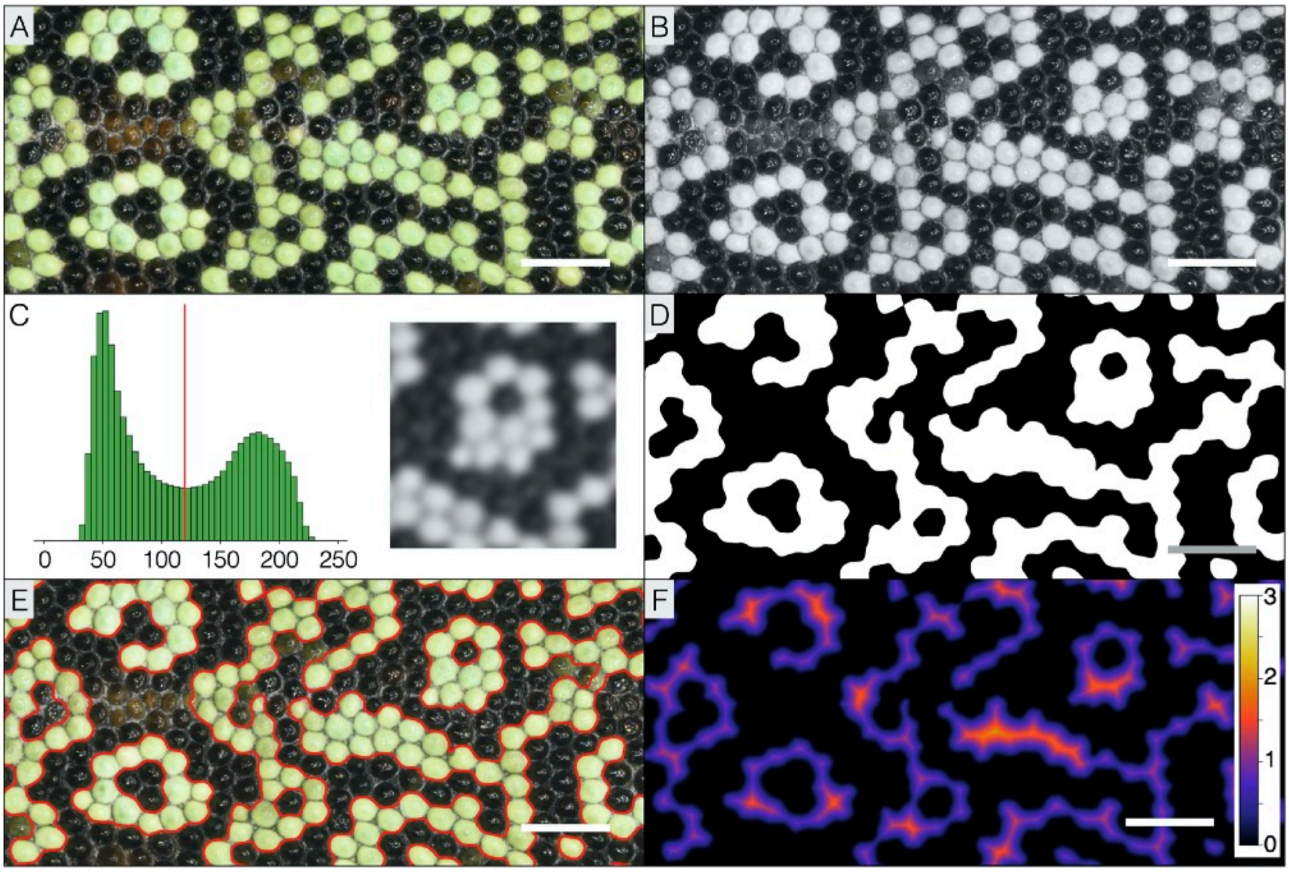

Supplementary Figure 4 | Pattern length scale quantification — (**A**) 2D image of the dorsal skin patch of a 45 week-old ocellated lizard. (**B**) Grayscale version of the image in A. (**C**) Histogram of grayscale intensities after blurring the image (inset) with a Gaussian filter. A 2-means clustering algorithm separates (red vertical line) the light and dark pixels. (**D**) Binary image resulting from thresholding based on the histogram in C. (**E**) Red lines indicate borders of green stripes detected on the original image in A. (**F**) Pixels belonging to green stripes are coloured by their normalised distance (measured in the number of skin scale diameters) to the closest border. Scale bars: 2 mm.

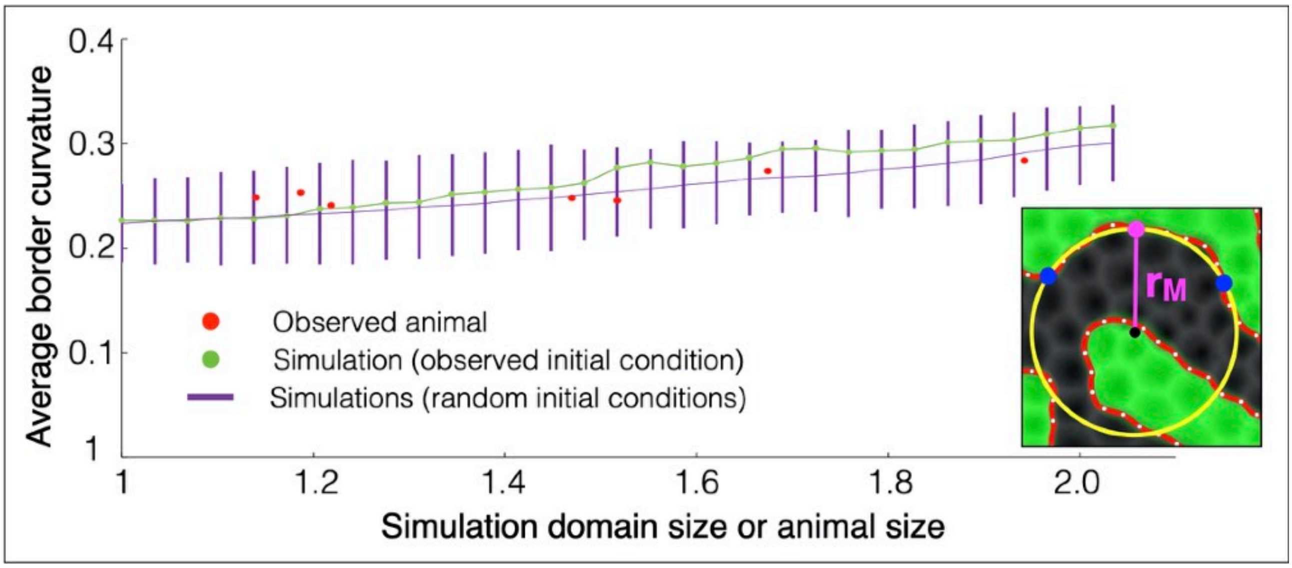

Supplementary Figure 5 I Growth affects patterning dynamics — Time evolution of average Menger curvature of the pattern border in a real animal and in a quasi-static growth simulation. Curvature ($1/r_M$) is computed, for points (white dots in inset) sampled every r_{avg} (*i.e.*, the average skin scale radius), along the border contour using neighbouring points (blue) that are $5.r_{avg}$ away from the point where the curvature is computed (purple). *I.e.*, curvature at the purple point is computed as the reciprocal of the radius (purple) of the circle (yellow) intercepting the purple and blue points. The curvature is normalised by r_{avg}.

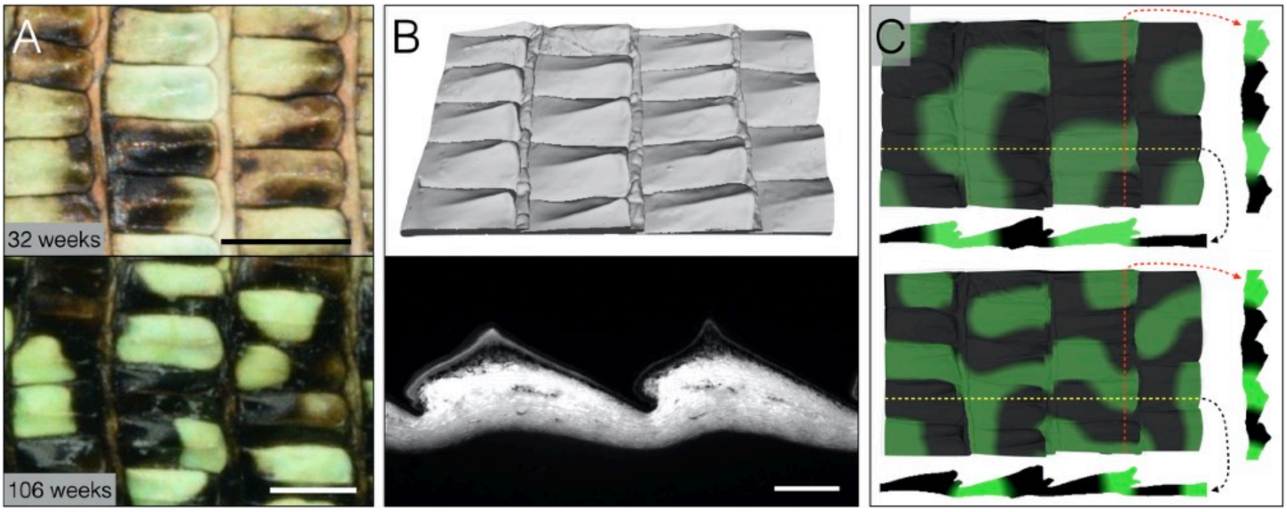

Supplementary Figure 6 I Patterns within tail scales — (**A**) Ocellated lizards tend to exhibit black and green patterns *within* tail scales as illustrated by an individual at the juvenile (32 week-old) and adult (106 week-old) stages. (**B**) Accurate 3D geometry of a 4x5 scales patch of tail skin (top panel) reconstructed with high-resolution episcopic microscopy sections (one section is displayed in the bottom panel). (**C**) Using the same RD model as in Figure 3E and 3F, simulations in 3D using the geometry reconstructed in (B) generates a pattern *within* scales both in a domain of size (ε=0.6; top panel) equivalent to a 32 week-old animal (initial condition = uniform steady state ±1% random noise), as well as after growth (using a quasi-static approach) to reach a size (ε=0.96; bottom panel) equivalent to a 106 week-old animal, *i.e.*, 60% larger than the 32 week-old juvenile. Yellow and red dotted lines in (C) indicate the position of transversal (red) and parasagittal (yellow) sections. Scale bars: 2 mm in (A) and 300 μm in (B).
