# Supplementary material for: Reaction-diffusion in a growing 3D domain of skin scales generates a discrete cellular automaton
Source: Nat Commun. 2021 Apr 23;12:2433. doi: 10.1038/s41467-021-22525-1 (PMC8065134; doi:10.1038/s41467-021-22525-1)
Supplement: Supplementary file 3 — Description of Additional Supplementary Files [file 41467_2021_22525_MOESM3_ESM.docx]

**Description of Additional Supplementary Files**

File Name: **Supplementary Movie 1** | **Growth affects scale-by-scale colour patterning in ocellated lizards.**

Description: The pattern mean relative length scale decreases as the animal grows. The dynamics observed in real lizards fit quantitatively the one predicted by numerical simulations.
