# Supplementary material for: Reaction-diffusion in a growing 3D domain of skin scales generates a discrete cellular automaton
Source: Nat Commun. 2021 Apr 23;12:2433. doi: 10.1038/s41467-021-22525-1 (PMC8065134; doi:10.1038/s41467-021-22525-1)
Supplement: Supplementary file 5 — Reporting Summary [file 41467_2021_22525_MOESM5_ESM.pdf]

nature research

Corresponding author(s): Michel C. Milinkovitch

Last updated by author(s): Mar 2, 2021

Reporting Summary

Nature Research wishes to improve the reproducibility of the work that we publish. This form provides structure for consistency and transparency in reporting. For further information on Nature Research policies, see our Editorial Policies and the Editorial Policy Checklist.

Statistics

For all statistical analyses, confirm that the following items are present in the figure legend, table legend, main text, or Methods section.

n/a	Confirmed	
☐	☒	The exact sample size (*n*) for each experimental group/condition, given as a discrete number and unit of measurement
☐	☒	A statement on whether measurements were taken from distinct samples or whether the same sample was measured repeatedly
☒	☐	The statistical test(s) used AND whether they are one- or two-sided<br>*Only common tests should be described solely by name; describe more complex techniques in the Methods section.*
☒	☐	A description of all covariates tested
☐	☒	A description of any assumptions or corrections, such as tests of normality and adjustment for multiple comparisons
☐	☒	A full description of the statistical parameters including central tendency (e.g. means) or other basic estimates (e.g. regression coefficient) AND variation (e.g. standard deviation) or associated estimates of uncertainty (e.g. confidence intervals)
☒	☐	For null hypothesis testing, the test statistic (e.g. *F*, *t*, *r*) with confidence intervals, effect sizes, degrees of freedom and *P* value noted<br>*Give P values as exact values whenever suitable.*
☒	☐	For Bayesian analysis, information on the choice of priors and Markov chain Monte Carlo settings
☒	☐	For hierarchical and complex designs, identification of the appropriate level for tests and full reporting of outcomes
☒	☐	Estimates of effect sizes (e.g. Cohen's *d*, Pearson's *r*), indicating how they were calculated

Our web collection on statistics for biologists contains articles on many of the points above.

Software and code

Policy information about availability of computer code

Data collection	Not computer code was used for data collection.
Data analysis	All of the code used for simulations have been deposited on Github at https://github.com/LANEvol/RD-3D.git

For manuscripts utilizing custom algorithms or software that are central to the research but not yet described in published literature, software must be made available to editors and reviewers. We strongly encourage code deposition in a community repository (e.g. GitHub). See the Nature Research guidelines for submitting code & software for further information.

Data

Policy information about availability of data

All manuscripts must include a data availability statement. This statement should provide the following information, where applicable:

- Accession codes, unique identifiers, or web links for publicly available datasets
- A list of figures that have associated raw data
- A description of any restrictions on data availability

The data generated or analysed during this study are included in this published article and its supplementary information file. Very large files with lizard 3D geometries are available from the corresponding author (M.C.M.) on reasonable request. All simulations can be replicated using the provided code.

Field-specific reporting

Life sciences study design

All studies must disclose on these points even when the disclosure is negative.

Sample size	Data are produced by numerical simulations for which the parameters are provided in the manuscript. Fig. 3C includes over 250'000 data points of raw data from 4 black and 4 green skin scales from a biopsy on a young adult male. Only Fig. 5 includes replicates: the pattern mean length scale is averaged among 20 independent simulations starting form different random initial conditions ; error bars = 3 standard deviation intervals.
Data exclusions	No data was excluded.
Replication	Replication of pattern formation has been verified by running multiple simulations with ±1% random noise
Randomization	No randomization of samples is required. Numerical simulations are preformed with a random seed and initial condition is perturbed with ±1% random noise.
Blinding	Blinding is not applicable to numerical simulations.

Reporting for specific materials, systems and methods

We require information from authors about some types of materials, experimental systems and methods used in many studies. Here, indicate whether each material, system or method listed is relevant to your study. If you are not sure if a list item applies to your research, read the appropriate section before selecting a response.

Materials & experimental systems

n/a	Involved in the study	
☒	☐	Antibodies
☒	☐	Eukaryotic cell lines
☒	☐	Palaeontology and archaeology
☐	☒	Animals and other organisms
☒	☐	Human research participants
☒	☐	Clinical data
☒	☐	Dual use research of concern

Methods

n/a	Involved in the study	
☒	☐	ChIP-seq
☒	☐	Flow cytometry
☒	☐	MRI-based neuroimaging

Animals and other organisms

Policy information about studies involving animals; ARRIVE guidelines recommended for reporting animal research

Laboratory animals	Lizards of the species Timon lepidus. One male of age 104 weeks for Figs. 1&5, Suppl. Figs 4&6. One biopsy from a male for Fig. 3A-C.
Wild animals	No wild animal were used in the study.
Field-collected samples	No field-collected samples were used in the study.
Ethics oversight	Maintenance of, and experiments on animals were approved by the Geneva Canton ethical regulation authority (authorisations GE/82/14, GE/73/16 AND GE/27/19) and performed according to Swiss law. These guidelines meet international standards.

Note that full information on the approval of the study protocol must also be provided in the manuscript.
